# Association of Knee Extensor Muscle Strength and Cardiorespiratory Fitness With Bone Stiffness in Japanese Adults: A Cross-sectional Study

**DOI:** 10.2188/jea.JE20200581

**Published:** 2022-12-05

**Authors:** Takahisa Ohta, Junzo Nagashima, Wataru Fukuda, Hiroyuki Sasai, Naokata Ishii

**Affiliations:** 1 Graduate School of Health and Sport Science, Nippon Sport Science University, Tokyo, Japan; 2 Yokohama Sports Medical Center, Nissan Stadium, Kanagawa, Japan; 3 Research Team for Promoting Independence and Mental Health, Tokyo Metropolitan Institute of Gerontology, Tokyo, Japan; 4 Division of Cardiology, Department of Internal Medicine, St. Marianna University School of Medicine, Kanagawa, Japan; 5 Department of Life Sciences, Graduate School of Arts and Sciences, The University of Tokyo, Tokyo, Japan

**Keywords:** knee extensor muscle strength, cardiorespiratory fitness, bone stiffness, quantitative ultrasound

## Abstract

**Background:**

Knee extensor muscle strength and cardiorespiratory fitness (CRF) are major components of physical fitness. Because the interactive association of knee extensor muscle strength and CRF with bone health remains unclear, we aimed to investigate such association in Japanese adults.

**Methods:**

Altogether, 8,829 Japanese adults (3,731 men and 5,098 women) aged ≥45 years completed the maximum voluntary knee extension test, submaximal exercise test, medical examination, and a questionnaire on lifestyle habits. Using an osteo-sono assessment index, low bone stiffness tendency was defined as 80% under the young-adults mean. Multivariable odds ratios (ORs) and 95% confidence intervals (CIs) were calculated after confounder adjustment.

**Results:**

Overall, 542 men (14.5%) and 978 women (19.2%) had low bone stiffness tendency. We observed an inverse association between muscle strength and low bone stiffness tendency after adjustment for CRF in both sexes (*P* for linear trend <0.001). Compared with the lowest CRF, the multivariable ORs for low bone stiffness tendency in the highest CRF were 0.47 (95% CI, 0.36–0.62) for men and 1.05 (95% CI, 0.82–1.35) for post-menopausal women (*P* < 0.001 and *P* = 0.704, respectively). No interactive association between muscle strength and CRF for low bone stiffness tendency existed in both sexes and irrespective of menopausal status.

**Conclusion:**

Knee extensor muscle strength and CRF were associated additively, not synergistically, with bone health. Maintaining high levels of both physical fitness components may improve musculoskeletal health in the cohort. The relationship between physical fitness and bone status should be longitudinally investigated in the future.

## INTRODUCTION

Osteoporosis is characterized by low bone strength and an increased risk of bone fractures,^1^ which is highly associated with mortality.^2^^,^^3^ Moreover, as osteoporosis imposes a heavy economic burden on the gross domestic product,^4^ it is widely recognized as a serious public health concern not only in Japan but also in other aged societies. Currently, Japan experiences one of the most serious situations in Asia^5^ because the estimated number of patients with osteoporosis exceeds 12.8 million (men: 3 million; women: 9.8 million).^6^ To minimize the detrimental effects of osteoporotic fractures on a patient’s quality of life, early detection of low bone strength and preventive interventions preferably based on risk stratification are strongly needed.

The established risk factors for low bone strength are advanced age, female sex, genetic factors, low body weight, and physical inactivity, including low physical fitness represented as cardiorespiratory fitness (CRF) and/or grip strength.^7^^,^^8^ Compared to low CRF, high CRF represents an odds ratio of 0.29 (95% confidence interval [CI], 0.12–0.71) for the femoral neck T-score ≤ 2.5.^7^ The hazard ratio for osteoporotic fracture per 5 kg reduction in hand grip strength was 1.49 (95% CI, 1.18–1.95) in a 10-year prospective cohort study.^8^ Maintaining high levels of physical fitness, assessed via CRF and/or hand grip strength, is a key factor for preventing low bone strength, which consequently lowers the risk of osteoporosis development.

Grip strength is often used as an indicator of muscle strength because it is valid and reliable for whole-body muscle strength.^9^ However, its use is controversial since it cannot be measured for antigravity muscles, such as the knee extensor muscle.^10^ As knee extensor muscle strength predicts the risk of falls and mobility, it can be used as a better indicator of bone strength.^11^^,^^12^ To our knowledge, it remains unclear whether knee extensor muscle strength is beneficially associated with bone strength. Moreover, CRF is also one of the predictors for low bone strength.^7^ However, information on the association between knee extensor muscle strength and bone strength according to varying levels of CRF is limited.

This study aimed to investigate the interactive associations of knee extensor muscle strength and CRF with bone stiffness as a marker of bone strength in Japanese adults. The findings from this cross-sectional study will allow us to design future longitudinal and clinical studies to investigate the preventive effect of various strategies for osteoporosis and possibly result in a decrease in osteoporotic fractures.

## METHODS

### Design, setting, and participants

This study was a cross-sectional analysis on the association of knee extensor muscle strength and CRF with bone stiffness. The eligibility criteria were: i) Sport Program Service (SPS, explained below) participants between April 1998 to July 2019, ii) aged ≥45 years, and iii) participants who had undergone bone stiffness measurements. SPS is a comprehensive medical checkup program primarily examining various domains of physical fitness held at the Yokohama Sports Medical Center. The SPS was initiated in April 1998 to improve the health status of people living or working in Yokohama City. Participants voluntarily apply to the service through the center’s website, and public information is published by the local government. This service receives an average of 10 people daily, totaling to 1,500 people annually. Participants eligible to use the service are those aged between 18 and 65 years, living or working in Yokohama City, and paid 15,000 JPY (approximately $142 in 2020); aged >65 years and paid 7,500 JPY ($71); and not living and working in Yokohama City, aged <65 years old, and paid 17,000 JPY ($161) and 8,500 JPY ($80). All data were selected at a single timepoint when participants first joined the service.

Prior to joining the SPS, all participants provided their informed consent for data use. This study was conducted according to the Declaration of Helsinki, and the protocol was approved by the Ethics Committee of the Yokohama Sports Medical Center (K-2019-07).

### Measurements

#### Medical checkup

Height and body weight of barefoot participants were measured using a calibrated height-weight scale (WB-510; Tanita Co., Tokyo, Japan). Body mass index (BMI) was calculated as body weight/height^2^ (kg/m^2^). After a 5-minute sitting position on a chair and medical examination by a physician, resting blood pressure was measured through the Riva-Rocci Korotkov method using a mercury sphygmomanometer. Blood glucose, total cholesterol, high-density lipoprotein (HDL) cholesterol, low-density lipoprotein (LDL) cholesterol, triglycerides (TG), alkaline phosphatase (ALP), and uric acid (UA) were sampled after 12-hour fasting and analyzed with Roche INTEGRA 400 plus (Roche International Ltd., Basle, Switzerland). Data on habitual alcohol drinking (yes or no), habitual smoking status (yes or no), and menopausal status (yes or no) were obtained through a self-reported questionnaire.

#### Bone stiffness and definition of low bone stiffness tendency

The generally known method for assessing bone strength is dual energy X-ray absorptiometry (DXA), which is commonly used to diagnose osteoporosis. As DXA emits radiation to assess the bone, there are problems, such as radiation exposure, costliness, being time-consuming, access to the place where DXA is set up, and the need for radiologists. In contrast, quantitative ultrasound (QUS), which is used to assess bone stiffness as an objective marker of bone strength, is well established in Japan.^6^ Compared to DXA, QUS results in better outcomes for the patients as it avoids exposure to radiation and is inexpensive, highly portable, and efficient. Therefore, in this study, bone stiffness was assessed using a QUS device (AOS-100NW; Hitachi Aloka Medical, Ltd., Mitaka, Tokyo, Japan) that has a high correlation with other QUS devices or DXA (*r* = 0.804, *P* < 0.001).^13^^,^^14^ Maintenance and calibration were performed to preserve the quality of the results once per month. The method of measurement was as follows: the participants sat on a chair barefoot with their knees bent at 90°. A device was placed on their right calcaneal, and gel was applied on the membranes. The measurement was performed once for approximately 30 seconds. Bone stiffness was expressed using the osteo-sono assessment index, which was calculated using the speed of sound and transmission index^15^ and was highly reproducible.^13^ Young adult mean (YAM) was calculated based on average age of 20 to 44 years as 100%.^16^ Low bone stiffness tendency was defined as 80% under the YAM (YAM80%), according to the 2011 Japanese guideline for prevention and treatment of osteoporosis—executive summary.^6^ Similarly, a 70% under the YAM (YAM70%) was also calculated to analyze sensitivity.

#### Physical fitness test

Knee extensor muscle strength, which assessed maximum voluntary knee extension torque, was measured using an isokinetic dynamometer (Cybex Humac Norm 770; Computer Sports Medicine Inc., Stoughton, MA, USA). The measurement process was as follows: the participants sat so that their knee and hip joint formed a right angle and performed warm-ups. Thereafter, the participants exerted isokinetic maximum voluntary knee extension at 60 degrees/s at least twice. Overall, the participants performed three repetitions with a 30-second break. The maximum value was considered as knee extensor strength (Nm) and adjusted by the participant’s own body weight (Nm/kg).

CRF was assessed by applying physical working capacity at 75% of the maximum heart rate (PWC75%HR_max_),^17^ which was highly correlated with maximal oxygen uptake (*r* = 0.942),^18^ using the submaximal graded exercise test method on an electronic bicycle ergometer (The Multi Exercise Test System, ML-1800, Fukuda-Denshi, Tokyo, Japan). On the graded exercise test, the rate of loading (10–60 W/min), which was an individualized ramp protocol, was decided by experts based on the participants’ age and habitual aerobic exercise. The target heart rate was set at 75% of the estimated maximum heart rate (220 minus age), and the test was ended upon reaching target value. Additionally, the participants could end the test when an abnormal electrocardiogram result (ST depression or frequent occurrence of the extrasystole) was confirmed by the cardiologists or when they could not pedal with the designated rhythm (50 rpm) while showing poor physical condition. Most participants ended the test at approximately 10 min.

### Statistical analysis

Due to clear sex and age differences in the prevalence of osteoporosis,^19^ the participants were segregated by sex and age group (aged 45–54, 55–64, and ≥65 years), and categorized into tertiles based on knee extensor muscle strength. Thereafter, each age category was combined based on muscle strength, and new groups were created with age-adjusted tertiles. Continuous and categorical variables were expressed as median (interquartile rages) or mean (standard deviation), and percentage, respectively.

To reduce potential biases due to incomplete data,^20^ missing data were treated with multiple imputation methods using SPSS (IBM, Inc., Chicago, IL, USA) by creating a random number through the Markov chain Monte Carlo algorithm, wherein 20 para-complete datasets were produced.^21^ Blood glucose, total cholesterol, HDL cholesterol, LDL cholesterol, TG, ALP, and UA were used as auxiliary variables to account for the missing data. The auxiliary variables were not used in the main analysis. The 20 datasets were integrated with the standard Rubin’s technique. Every missing value is presented in Table 1.

**Table 1.** Participant characteristics according to knee extensor muscle strength

		Men					Women				

		Overall (*n* = 3,731)	Lowest (*n* = 1,245)	Middle (*n* = 1,243)	Highest (*n* = 1,243)	Missing, *n* (%)	Overall (*n* = 5,098)	Lowest (*n* = 1,699)	Middle (*n* = 1,700)	Highest (*n* = 1,699)	Missing, *n* (%)
**Age**	year	62.0 [15.0]	62.0 [16.0]	62.0 [15.0]	61.0 [14.0]	—	59.0 [14.0]	64.0 [12.0]	59.0 [13.0]	54.0 [12.0]	
**Knee extensor muscle strength**	Nm/kg	2.13 [0.61]	1.70 [0.47]	2.11 [0.39]	2.58 [0.44]	—	1.59 [0.46]	1.26 [0.25]	1.59 [0.14]	1.93 [0.28]	
**Cardiorespiratory fitness**	w/kg	1.64 [0.66]	1.51 [0.61]	1.58 [0.58]	1.78 [0.68]	1,475 (39.5)	1.30 [0.52]	1.13 [0.49]	1.25 [0.43]	1.48 [0.52]	2,102 (41.2)
**Height**	cm	167.4 [8.0]	166.8 [8.1]	167.5 [8.0]	167.8 [7.8]	—	155.2 [7.6]	153.8 [7.3]	155.3 [7.5]	156.5 [7.4]	—
**Body weight**	kg	64.8 [11.3]	66.7 [12.5]	65.0 [11.2]	62.8 [9.9]	—	53.0 [11.3]	55.0 [12.2]	53.1 [11.0]	51.2 [9.6]	—
**Body mass index**	kg/m^2^	23.1 [3.4]	23.8 [3.7]	23.2 [3.3]	22.4 [3.0]	—	22.1 [4.3]	23.2 [4.7]	22.2 [4.0]	21.0 [3.5]	—
**Blood glucose**	mg/dL	103.0 [15.0]	104.0 [16.0]	103.0 [15.0]	102.0 [13.0]	27 (0.7)	98.0 [13.0]	98.0 [15.0]	98.0 [13.0]	97.0 [11.0]	36 (0.7)
**Total cholesterol**	mg/dL	211.0 [42.0]	211.0 [43.0]	210.0 [41.0]	214.2 (30.8)	27 (0.7)	226.0 [45.0]	228.0 [45.0]	227.0 [45.0]	224.0 [45.0]	36 (0.7)
**HDL cholesterol**	mg/dL	56.0 [21.0]	53.0 [19.0]	55.0 [19.0]	60.0 [21.0]	27 (0.7)	66.0 [22.0]	64.0 [21.0]	66.0 [23.0]	69.0 [22.0]	36 (0.7)
**LDL cholesterol**	mg/dL	123.0 [38.0]	125.0 [39.0]	123.0 [39.0]	120.0 [36.0]	27 (0.7)	130.0 [41.0]	134.0 [41.0]	131.0 [41.0]	123.0 [40.0]	36 (0.7)
**Triglyceride**	mg/dL	99.0 [68.0]	110.0 [75.0]	103.0 [69.0]	87.0 [58.0]	27 (0.7)	82.0 [51.0]	92.0 [54.0]	84.0 [52.0]	71.0 [44.0]	36 (0.7)
**Alkaline phosphatase**	IU/mL	185.0 [76.0]	179.0 [81.0]	183.0 [77.5]	192.5 [70.0]	143 (3.8)	195.0 [93.0]	197.0 [97.0]	196.0 [92.3]	192.0 [89.0]	477 (9.4)
**Uric acid**	mg/dL	5.8 [1.5]	5.8 (1.2)	5.8 [1.4]	5.7 [1.6]	27 (0.7)	4.4 [1.3]	4.5 [1.3]	4.4 [1.3]	4.3 [1.3]	36 (0.7)
**Systolic blood pressure**	mm Hg	120.0 [22.0]	122.0 [22.0]	122.0 [20.0]	120.0 [20.0]	1 (0.0)	120.0 [20.0]	120.0 [22.0]	120.0 [20.0]	116.0 [24.0]	1 (0.0)
**Diastolic blood pressure**	mm Hg	70.0 [18.0]	70.0 [16.0]	70.0 [16.0]	70.0 [20.0]	1 (0.0)	70.0 [20.0]	70.0 [18.0]	70.0 [20.0]	68.0 [12.0]	1 (0.0)
**Menopause**	*n* (%)	—	—	—	—	—	3,844 (79.7)	1,450 (91.0)	1,299 (80.7)	1,095 (67.6)	276 (5.4)
**Smoking status**	*n* (%)	577 (15.5)	254 (20.4)	187 (15.1)	136 (11.0)	12 (0.3)	254 (5.0)	83 (4.9)	81 (4.8)	90 (5.3)	18 (0.4)
**Drinking habits**	*n* (%)	2,554 (68.4)	812 (65.5)	858 (69.2)	874 (70.5)	12 (0.3)	1,418 (27.9)	354 (21.0)	479 (28.3)	585 (34.5)	23 (0.5)
**OSI**	×10^6^	2.85 [0.45]	2.79 [0.42]	2.85 [0.44]	2.92 [0.43]		2.48 [0.38]	2.40 [0.32]	2.48 [0.36]	2.58 [0.44]	
**<70% YAM**	*n* (%)	45 (1.2)	26 (2.1)	12 (1.0)	7 (0.6)		49 (1.0)	28 (1.6)	14 (0.8)	7 (0.4)	
**<80% YAM**	*n* (%)	542 (14.5)	246 (19.8)	175 (14.1)	121 (9.7)		978 (19.2)	434 (25.5)	319 (18.8)	225 (13.2)	

HDL, high-density lipoprotein; LDL, low-density lipoprotein cholesterol; OSI, osteo-sono assessment index; and YAM, young-adult mean.

Data are presented as median [interquartile ranges], mean (standard deviation), and number (percentage) unless specified.

To assess the association of each covariate, knee extensor muscle strength, and CRF with bone stiffness, univariable odds ratios and 95% CIs were calculated using a logistic regression model. The covariates included age,^22^ smoking status,^23^ drinking habits,^24^ BMI,^25^ systolic blood pressure,^26^ and menopause.^23^

To evaluate the association between knee extensor muscle strength and low bone stiffness tendency, multivariable odds ratios and 95% CIs were calculated using the lowest knee extensor muscle strength group as reference after adjusting for smoking status, drinking habits, BMI, systolic blood pressure, and menopause (for women only). In the mutually adjusted final model, CRF was entered into the final model. Moreover, a continuous variable of knee extensor muscle strength was used in another model to test linearity. The same analyses were repeated with CRF as the primary exposure and knee extensor muscle strength as the final covariate. To consider the moderating effect of menopausal status, a menopause-stratified multivariable analysis was also performed.

To evaluate the interactive association of knee extensor muscle strength and CRF with low bone stiffness tendency, a CRF-stratified (below or above the median) multivariable analysis was performed. Odds ratios and 95% CIs were calculated similarly as mentioned above, and the interaction term (knee extensor muscle strength*CRF) was entered into the models. A menopause-stratified multivariable analysis was also conducted.

To verify the validity of the applicable standards for low bone stiffness tendency, the following two sensitivity analyses were performed. First, we repeated the primary analysis by changing the definition of low bone stiffness tendency from 80% under the YAM to 70% under the YAM. Second, a complete-case analysis was conducted to distinguish findings with and without considering missing values. All sensitivity analysis data are shown in the eTable 1, eTable 2, eTable 3, eTable 4, eTable 5, eTable 6, and eTable 7.

All statistical analyses were performed using the SPSS version 25. A *P*-value <0.05 was considered significant.

## RESULTS

**Figure 1.**
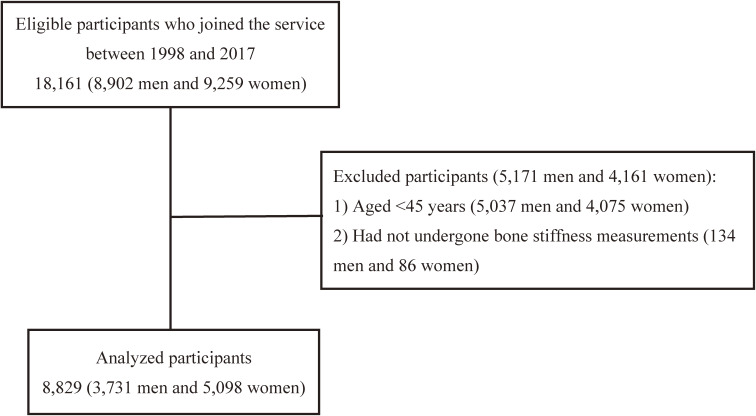
Flowchart of participant selection

Altogether, 18,161 adults (8,902 men and 9,259 women) joined the service from April 1998 to July 2017. Of them, 9,112 aged ≤45 years old and 220 without bone stiffness measurements were excluded (Figure 1). Of the final sample of 8,829 (3,731 men and 5,098 women), 542 men (14.5%) and 978 women (19.2%) had low bone stiffness tendency based on YAM80%. Similarly, 45 men (1.2%) and 49 women (1.0%) were classified as having low bone stiffness based on YAM70%. The lowest fitness category appears to have an inferior biomarker status, such as triglycerides than the other categories (Table 1).

Table 2 shows the association between each potential covariate and bone stiffness. The association between smoking status and low bone stiffness tendency was found in men (*P* < 0.001), but not in women (*P* = 0.097). Low BMI, low knee extensor muscle strength, and low CRF were all associated with a higher prevalence of low bone stiffness tendency.

**Table 2.** Univariate associations of each covariate and two main exposure variables with low bone stiffness tendency

Variables	Units	Men					Women				

		Participant	Frequency	Prevalence^a^	Odds ratio (95% CI)	*P* value	Participant	Frequency	Prevalence^a^	Odds ratio (95% CI)	*P* value
**Age**	Years	3,731	542	145.3	1.02 (1.01–1.03)	<0.001	5,098	978	191.8	1.10 (1.09–1.11)	<0.001
**Smoking status**	No	3,153	422	133.8	1.00 (reference)	<0.001	4,844	930	192.0	1.00 (reference)	0.907
	Yes	578	120	207.6	1.70 (1.35–2.13)		254	48	189.0	0.98 (0.71–1.35)	
**Drinking habits**	No	1,180	202	171.2	1.00 (reference)	0.002	3,674	779	212.0	1.00 (reference)	<0.001
	Yes	2,551	340	133.3	0.74 (0.61–0.90)		1,424	199	139.7	0.61 (0.51–0.72)	
**Body mass index**	<18.5	109	34	311.9	2.58 (2.08–3.20)	0.029	448	141	318.3	1.89 (1.53–2.35)	<0.001
	18.5–25.0	2,704	405	149.8	1.00 (reference)		3,602	712	197.9	1.00 (reference)	
	≥25.0	908	103	113.4	0.73 (0.65–0.82)		1,053	122	116.1	0.53 (0.43–0.65)	
**Menopause**	No	—	—		—	—	1,039	19	18.3	1.00 (reference)	<0.001
	Yes	—	—		—	—	4,060	959	236.2	16.33 (10.31–25.88)	
**Knee extensor ** **muscle strength**	Lowest	1,245	246	197.6	1.00 (reference)	<0.001	1,699	434	255.4	1.00 (reference)	<0.001
	Middle	1,243	175	140.8	0.67 (0.54–0.82)		1,700	319	187.6	0.67 (0.57–0.79)	
	Highest	1,243	121	97.3	0.44 (0.35–0.55)		1,699	225	132.4	0.45 (0.37–0.53)	
**Cardiorespiratory fitness**	Lowest	1,243	222	178.6	1.00 (reference)	<0.001	1,699	375	220.7	1.00 (reference)	<0.001
	Middle	1,245	172	138.2	0.78 (0.60–1.00)		1,698	326	192.0	0.77 (0.63–0.95)	
	Highest	1,243	148	119.1	0.58 (0.45–0.78)		1,701	277	162.8	0.68 (0.55–0.84)	

CI, confidence interval.

^a^Prevalence per 1,000 persons.

An inverse association between knee extensor muscle strength and low bone stiffness tendency was observed after adjusting for potential confounders, such as age, smoking status, drinking habits, BMI, systolic blood pressure, menopause (for women only), and CRF in both sexes (*P* for linear trend <0.001 for both). Meanwhile, CRF and low bone stiffness tendency showed an inverse association in men (*P* for linear trend <0.001), but not in pre-menopausal, post-menopausal, and all women (*P* for linear trend = 0.634, 0.841, and 0.924, respectively), as shown in Table 3. Both knee extensor muscle strength and CRF had no interactive association with low bone stiffness tendency in both sexes (men; *P* = 0.836, all women; *P* = 0.700, post-menopausal women; *P* = 0.615), as shown in Table 4.

**Table 3.** Associations of knee extensor muscle strength or cardiorespiratory fitness with low bone stiffness tendency

	Knee extensor muscle strength			*P* for trend

	Lowest	Middle	Highest	
**Men**				
Age adjusted	1.00 (reference)	0.69 (0.55–0.84)	0.45 (0.36–0.57)	<0.001
Multivariable adjusted^a^	1.00 (reference)	0.65 (0.54–0.79)	0.41 (0.36–0.46)	<0.001
Mutual adjusted^b^	1.00 (reference)	0.65 (0.52–0.81)	0.42 (0.32–0.53)	<0.001
**Women**				
Age adjusted	1.00 (reference)	0.94 (0.79–1.12)	0.86 (0.71–1.05)	0.145
Multivariable adjusted^a^	1.00 (reference)	0.79 (0.66–0.95)	0.62 (0.50–0.76)	<0.001
Mutual adjusted^b^	1.00 (reference)	0.79 (0.66–0.95)	0.62 (0.50–0.77)	<0.001
Pre-menopause				
Age adjusted	1.00 (reference)	0.35 (0.09–1.35)	0.49 (0.15–1.55)	0.343
Multivariable adjusted^a^	1.00 (reference)	0.31 (0.08–1.20)	0.30 (0.09–1.02)	0.090
Mutual adjusted^b^	1.00 (reference)	0.30 (0.08–1.21)	0.31 (0.09–1.05)	0.100
Post-menopause				
Age adjusted	1.00 (reference)	0.95 (0.80–1.13)	0.89 (0.73–1.09)	0.258
Multivariable adjusted^a^	1.00 (reference)	0.81 (0.67–0.97)	0.63 (0.51–0.78)	<0.001
Mutual adjusted^b^	1.00 (reference)	0.80 (0.67–0.97)	0.63 (0.51–0.78)	<0.001

	Cardiorespiratory fitness			*P* for trend

	Lowest	Middle	Highest	

**Men**				
Age adjusted	1.00 (reference)	0.80 (0.61–1.02)	0.61 (0.46–0.81)	<0.001
Multivariable adjusted^a^	1.00 (reference)	0.74 (0.55–0.97)	0.45 (0.33–0.62)	<0.001
Mutual adjusted^b^	1.00 (reference)	0.75 (0.63–0.88)	0.47 (0.36–0.62)	<0.001
**Women**				
Age adjusted	1.00 (reference)	1.02 (0.81–1.28)	1.25 (0.98–1.61)	0.085
Multivariable adjusted^a^	1.00 (reference)	0.89 (0.70–1.12)	0.99 (0.77–1.23)	0.899
Mutual adjusted^b^	1.00 (reference)	0.91 (0.72–1.15)	1.02 (0.79–1.31)	0.924
Pre-menopause				
Age adjusted	1.00 (reference)	1.60 (0.33–7.69)	0.94 (0.16–5.74)	0.875
Multivariable adjusted^a^	1.00 (reference)	1.50 (0.29–8.16)	0.65 (0.10–4.28)	0.529
Mutual adjusted^b^	1.00 (reference)	1.69 (0.31–9.26)	0.74 (0.11–5.02)	0.632
Post-menopause				
Age adjusted	1.00 (reference)	0.96 (0.77–1.21)	1.23 (0.97–0.57)	0.111
Multivariable adjusted^a^	1.00 (reference)	0.93 (0.74–1.18)	1.03 (0.81–1.32)	0.851
Mutual adjusted^b^	1.00 (reference)	0.96 (0.76–1.21)	1.05 (0.82–1.35)	0.704

Values are expressed as odds ratio (95% confidence interval).

^a^Additionally adjusted smoking status, drinking habits, body mass index, systolic blood pressure, and menopause (for women only).

^b^Mutually adjusted cardiorespiratory fitness or knee extensor muscle strength plus variables in multivariable adjustment^a^.

**Table 4.** Interacting association of knee extensor muscle strength and cardiorespiratory fitness with low bone stiffness tendency

	Participant	Frequency	Prevalence^a^	Odds ratio (95% CI)^b^	Combined odds ratio (95% CI)^c^	*P* for interaction
**Men**						0.836
**Lowest** cardiorespiratory fitness						
**Lowest** knee extensor muscle strength	703	148	210.5	1.00 (reference)	1.00 (reference)	
**Middle** knee extensor muscle strength	680	112	164.7	0.76 (0.51–0.99)	0.71 (0.51–0.99)	
**Highest** knee extensor muscle strength	481	55	114.3	0.39 (0.26–0.58)	0.39 (0.27–0.58)	
**Highest** cardiorespiratory fitness						
**Lowest** knee extensor muscle strength	542	98	180.8	1.00 (reference)	0.70 (0.48–1.02)	
**Middle** knee extensor muscle strength	563	63	111.9	0.56 (0.54–0.87)	0.39 (0.26–0.58)	
**Highest** knee extensor muscle strength	762	66	86.6	0.44 (0.30–0.65)	0.31 (0.22–0.44)	

**Women**						0.700
**Lowest** cardiorespiratory fitness						
**Lowest** knee extensor muscle strength	1,037	267	257.5	1.00 (reference)	1.00 (reference)	
**Middle** knee extensor muscle strength	902	177	196.2	0.75 (0.58–0.96)	0.77 (0.60–1.00)	
**Highest** knee extensor muscle strength	612	96	156.9	0.61 (0.43–0.86)	0.66 (0.47–0.92)	
**Highest** cardiorespiratory fitness						
**Lowest** knee extensor muscle strength	663	161	242.8	1.00 (reference)	1.05 (0.76–1.46)	
**Middle** knee extensor muscle strength	798	145	181.7	0.86 (0.62–1.18)	0.85 (0.62–1.16)	
**Highest** knee extensor muscle strength	1,088	132	121.3	0.64 (0.45–0.91)	0.62 (0.46–0.83)	
Pre-menopause						0.373
**Lowest** cardiorespiratory fitness						
**Lowest** knee extensor muscle strength	79	4	50.6	1.00 (reference)	1.00 (reference)	
**Middle** knee extensor muscle strength	137	2	14.6	0.28 (0.01–7.37)	0.44 (0.03–7.16)	
**Highest** knee extensor muscle strength	170	1	5.9	0.05 (0.00–1.41)	0.12 (0.01–2.45)	
**Highest** cardiorespiratory fitness						
**Lowest** knee extensor muscle strength	70	3	42.9	1.00 (reference)	0.87 (0.04–20.44)	
**Middle** knee extensor muscle strength	195	2	10.3	0.05 (0.00–2.06)	0.04 (0.00–2.38)	
**Highest** knee extensor muscle strength	388	7	18.0	0.44 (0.06–3.12)	0.39 (0.03–4.48)	
Post-menopause						0.615
**Lowest** cardiorespiratory fitness						
**Lowest** knee extensor muscle strength	958	265	276.6	1.00 (reference)	1.00 (reference)	
**Middle** knee extensor muscle strength	765	180	235.3	0.76 (0.59–0.98)	0.78 (0.61–1.01)	
**Highest** knee extensor muscle strength	441	94	213.2	0.64 (0.45–0.90)	0.68 (0.49–0.96)	
**Highest** cardiorespiratory fitness						
**Lowest** knee extensor muscle strength	592	162	273.6	1.00 (reference)	1.05 (0.76–1.44)	
**Middle** knee extensor muscle strength	603	135	223.9	0.88 (0.64–1.22)	0.88 (0.65–1.19)	
**Highest** knee extensor muscle strength	701	123	175.5	0.64 (0.45–0.91)	0.62 (0.46–0.84)	

CI, confidence interval.

^a^Prevalence per 1,000 persons.

^b^Adjusted for age, smoking status, drinking habits, body mass index, systolic blood pressure, and menopause (for women only).

^c^Using the lowest cardiorespiratory fitness and the lowest knee extensor muscle strength as reference.

## DISCUSSION

Our primary findings were as follows. First, no interactive association between knee extensor muscle strength and CRF was found for low bone stiffness tendency in both sexes. Second, higher knee extensor muscle strength was associated with a lower prevalence of low bone stiffness tendency in both sexes, independent of CRF. Third, higher CRF was associated with a lower prevalence of low bone stiffness tendency in men but not in women, independent of knee extensor muscle strength. These findings suggest that a combination of muscle strength and CRF may carry an additive benefit, not synergistic, for bone health. However, the association between CRF and bone health may not be evident in adult Japanese women.

Contrary to our expectation, the knee extensor muscle strength and CRF had no interactive association with bone stiffness. Hence, each of the two physical fitness elements is independently or additively, not synergistically, associated with bone health. As described in the subsequent paragraphs, the possible mechanisms of muscle strength or CRF to bone strength may be different. However, it is unknown whether the two different pathways interfere with or work synergistically with each other, due to lack of findings from basic mechanistic studies. A previous randomized controlled trial has revealed that resistance exercise and a combination of resistance and aerobic exercise could prevent bone loss compared to aerobic exercise alone during a weight loss program.^27^ This finding supports the notion that a combined aerobic and resistance exercise may be recommended to prevent a loss of musculoskeletal function.^28^ This may be consistent with our results.

Knee extensor muscle strength was positively associated with bone stiffness after adjusting for CRF in both sexes. According to Wolff’s law, theories have indicated the potential of the bone to adapt its remodeling response to external stressors.^29^ It is assumed that knee extensor muscle strength may play the role of an external stressor to improve bone stiffness. Meanwhile, another possible mechanism of association between muscle strength and bone stiffness is related to muscle functioning as an endocrine organ that secretes myokines. Previous physiological studies have reported that muscles can play a role in secreting myokines that activate bone metabolism, such as myostatin, insulin-like growth factor (IGF)-1, and IGF-2.^30^ A previous observational study has reported an association between muscle strength and nonadjacent bones.^31^ However, the details of this association remain unclear, so further investigations into the mechanisms are warranted. Because bone mineral density has been highly correlated with bone stiffness,^13^^,^^14^ these are reflected in our study’s results, as knee extensor muscle strength was associated with bone stiffness after adjusting for confounders.

CRF was also deemed beneficial for bone health in men. A previous large-scale observational study has showed similar trends as this study.^32^ CRF partially reflects one’s physical activity level and has been utilized as an index of health outcome.^33^ Because physical activity produces mechanical loading to the musculoskeletal system, which enhances osteocyte activation and bone resorption and formation, the American College of Sport Medicine currently recommends weightbearing physical activities.^34^ This study identified a sex difference in the association between CRF and bone stiffness (ie, no association in women). From a biological viewpoint, one possible mechanism for bone loss could be greater age-dependent loss of estrogen in women, which reduces estrogen receptor and osteogenic response.^35^ Accordingly, no association between CRF and bone health was observed in women, as in a previous study.^36^

This study has several noteworthy strengths. First, we analyzed a large-scale population of participants across a wide age range (≥45 years old). A difference in the age of onset between men and women of osteoporosis caused by lowering bone strength was observed.^19^ The number of patients with osteoporosis drastically increases after menopause for women and after the age of 60 for men.^19^ Second, we reduced potential biases by using multiple imputation methods.^21^ Third, this study expanded the body of knowledge in this area by examining the joint associations between knee extensor muscle strength and CRF with bone stiffness. Fourth, we used an isokinetic knee extensor muscle strength dynamometer, which enabled precise measurements. Although it requires expert skills, isokinetic muscle strength that reflects active physical activity could be assessed with accuracy.^37^

Meanwhile, there are also several limitations. First, causal associations among knee extensor muscle strength, CRF, and bone stiffness could not be assessed owing to the cross-sectional nature of the study. Second, there was no information on habitual dietary intake, such as protein, which could positively contribute to bone mineral density.^38^ Thus, the risk of residual confounding by dietary habits may exist in the results. Third, there was a lack of information on the use of osteoporosis medication and the history of surgery. Hence, bone stiffness could be underestimated. Finally, there was a lack of information on habitual physical activity. As half of the variances in CRF are accounted for by one’s genotype, CRF is not a direct marker of physical activity, although it may reflect an aspect of physical activity.^39^ Therefore, precise assessments of physical activity are preferable.

### Conclusion

We found an additive association between knee extensor muscle strength and CRF for bone health. Moreover, an inverse association existed between knee extensor muscle strength and low bone stiffness tendency in both sexes. Accordingly, maintaining not only higher knee extensor muscle strength, but also higher CRF may be important to prevent or delay the onset of osteoporosis. From a practical viewpoint, these results suggest that middle-aged or older women and men should be encouraged to participate in high-intensity physical activities with mechanical loading, such as running, jumping rope, and resistance exercise. Further longitudinal studies are needed to investigate the relationship between physical fitness and bone status.

## SUPPLEMENTARY MATERIAL

eTable 1. Participant characteristics according to cardiorespiratory fitness

eTable 2. Univariate associations of each covariate and two main exposure variables with low bone stiffness (YAM70%).

eTable 3. Associations of knee extensor muscle strength or cardiorespiratory fitness with low bone stiffness (YAM70%)

eTable 4. Interacting association of knee extensor muscle strength and cardiorespiratory fitness with low bone stiffness (YAM70%)

eTable 5. Univariate associations of each covariate and two main exposure variables with low bone stiffness (complete case)

eTable 6. Associations of knee extensor muscle strength or cardiorespiratory fitness with low bone stiffness (complete case)

eTable 7. Interacting association of knee extensor muscle strength and cardiorespiratory fitness with low bone stiffness (complete case)
