# Supplementary material for: Association of Knee Extensor Muscle Strength and Cardiorespiratory Fitness With Bone Stiffness in Japanese Adults: A Cross-sectional Study
Source: J Epidemiol. 2022 Dec 5;32(12):543–50. doi: 10.2188/jea.JE20200581 (PMC9643791; doi:10.2188/jea.JE20200581)
Supplement: Supplementary file 1 [file je-32-543-s001.pdf]

eTable 1. Participant characteristics according to cardiorespiratory fitness

		Men					Women				
		**Overall (n=2,256)**	**Lowest (n=752)**	**Middle (n=750)**	**Highest (n=754)**	**Missing, n (%)**	**Overall (n=2,996)**	**Lowest (n=1,013)**	**Middle (n=983)**	**Highest (n=1,000)**	**Missing, n (%)**
**Age**	year	63.0 [14.0]	63.0 [15.0]	63.0 [14.0]	63.0 [14.0]	–	57.0 [14.0]	66.0 [12.0]	60.0 [12.0]	55.0 [11.0]	
**Knee extensor muscle strength**	Nm/kg	2.18 [0.62]	2.06 [0.64]	2.18 [0.55]	2.31 [0.62]	–	1.53 [0.45]	1.49 [0.41]	1.62 [0.41]	1.80 [0.47]	
**Cardiorespiratory fitness**	w/kg	1.64 [0.66]	1.18 [0.48]	1.64 [0.46]	2.11 [0.51]	–	1.30 [0.52]	0.93 [0.28]	1.30 [0.16]	1.68 [0.33]	–
**Height**	cm	167.5 [7.9]	167.5 [8.1]	167.7 [7.7]	167.3 [7.8]	–	155.1 [7.4]	154.1 [8.0]	155.4 [7.4]	156.2 [7.3]	–
**Body weight**	kg	64.2 [11.5]	67.4 [13.1]	64.8 [11.2]	61.6 [9.9]	–	53.4 [10.9]	54.3 [13.5]	53.3 [12.9]	51.0 [8.9]	–
**Body mass index**	kg/m^2	22.9 [3.4]	23.9 [3.8]	23.1 [3.3]	22.0 [2.7]	–	22.2 [4.0]	23.1 [5.0]	22.1 [4.4]	20.7 [3.4]	–
**Blood glucose**	mg/dL	103.0 [14.0]	107.0 [17.0]	103.0 [13.0]	101.0 [11.5]	21 (0.9)	96.0 [12.0]	102.0 [15.0]	99.0 [12.0]	96.0 [12.0]	26 (0.9)
**Total cholesterol**	mg/dL	212.0 [42.0]	214.0 [44.0]	213.0 [41.0]	211.4 (29.6)	21 (0.9)	224.0 [45.0]	231.0 [48.0]	230.3 (34.3)	224.0 [44.0]	26 (0.9)
**HDL cholesterol**	mg/dL	57.0 [21.0]	53.0 [18.0]	56.0 [19.0]	63.0 [23.0]	21 (0.9)	67.0 [23.0]	63.0 [21.0]	65.0 [20.0]	71.0 [23.0]	26 (0.9)
**LDL cholesterol**	mg/dL	120.0 [37.0]	123.0 [39.0]	121.6 (26.4)	115.8 (25.6)	21 (0.9)	135.0 [43.0]	131.0 [41.0]	129.0 [39.0]	119.0 [38.0]	26 (0.9)
**Triglyceride**	mg/dL	94.0 [64.5]	109.0 [76.3]	99.0 [68.5]	78.0 [53.0]	21 (0.9)	82.0 [51.0]	95.0 [58.0]	86.0 [52.0]	69.0 [38.0]	26 (0.9)
**Alkaline phosphatase**	IU/mL	201.0 [70.0]	205.0 [80.0]	202.0 [69.8]	195.0 [65.0]	126 (5.6)	164.0 [77.0]	232.0 [81.0]	222.0 [82.0]	204.0 [86.3]	433 (14.5)
**Uric acid**	mg/dL	5.8 [1.7]	6.0 [1.5]	5.8 [1.5]	5.4 [1.6]	21 (0.9)	4.2 [1.3]	4.8 [1.5]	4.6 [1.2]	4.3 [1.3]	26 (0.9)

**Systolic blood pressure**	mmHg	120.0 [20.0]	120.0 [18.0]	120.0 [20.0]	119.0 [14.0]	1 (0.0)	122.0 [25.0]	120.0 [20.0]	118.0 [14.0]	110.0 [20.0]	–
**Diastolic blood pressure**	mmHg	70.0 [16.0]	70.0 [16.0]	70.0 [18.0]	70.0 [10.0]	1 (0.0)	70.0 [18.0]	70.0 [16.0]	68.0 [10.0]	62.0 [10.0]	–
**Menopause**	n (%)	–	–	–	–	–	2382 (79.5)	894 (88.3)	797 (81.1)	691 (69.1)	106 (3.5)
**Smoking status**	n (%)	168 (7.4)	80 (10.6)	87 (11.6)	86 (11.4)	3 (0.1)	106 (3.5)	18 (1.8)	26 (2.6)	62 (6.2)	6 (0.2)
**Drinking habits**	n (%)	1520 (67.4)	470 (62.5)	521 (69.5)	529 (70.2)	5 (0.2)	912 (30.4)	221 (21.8)	288 (29.3)	403 (40.3)	5 (0.2)
**OSI**	$\times 10^6$	2.87 [0.44]	2.83 [0.41]	2.88 [0.42]	2.92 [0.47]		2.47 [0.38]	2.43 [0.36]	2.48 [0.38]	2.56 [0.44]	
**< 70% YAM**	n (%)	23 (1.0)	12 (1.6)	7 (1.0)	4 (0.5)		30 (1.0)	13 (1.3)	11 (1.1)	6 (0.6)	
**< 80% YAM**	n (%)	244 (10.8)	106 (14.1)	82 (10.9)	56 (7.4)		527 (17.6)	217 (21.4)	182 (18.5)	128 (12.8)	

HDL, high-density lipoprotein; LDL, low-density lipoprotein cholesterol; OSI, osteo-sono assessment index; YAM, young-adult mean.
Data were presented as median [interquartile ranges], mean (standard deviation) and number (percentage) otherwise specified.

eTable 2. Univariate associations of each covariate and two main exposure variables with low bone stiffness (YAM70%)

		Men					Women				
**Variables**	**Units**	**Participant**	**Frequency**	**Prevalence**[a]	**Odds ratio (95% CI)**	***P* value**	**Participant**	**Frequency**	**Prevalence**[a]	**Odds ratio (95% CI)**	***P* value**
**Age**	Years	3,731	45	12.1	1.08 (1.04–1.12)	<0.001	5,098	49	9.6	1.17 (1.12–1.21)	<0.001
**Smoking status**	No	3,153	32	10.1	1.00 (reference)	<0.001	4,844	48	9.9	1.00 (reference)	0.377
	Yes	578	13	22.5	2.24 (1.61–3.13)		254	1	3.9	0.41 (0.06–2.98)	
**Drinking habits**	No	1,180	17	14.4	1.00 (reference)	0.373	3,674	43	11.7	1.00 (reference)	0.027
	Yes	2,551	28	11.0	0.76 (0.43–1.35)		1,424	6	4.2	0.38 (0.16–0.90)	
**Body mass index**	<18.5	109	12	110.1	10.69 (7.43–15.39)	0.206	448	16	36.1	4.61 (2.48–8.56)	0.931
	18.5–25.0	2,704	31	11.4	1.00 (reference)		3,602	29	8.1	1.00 (reference)	
	≥25.0	908	2	2.2	0.19 (0.09–0.40)		1,053	4	3.8	0.47 (0.17–1.34)	
**Menopause**	No	–	-	-	-	-	1,039	0	0	-	-
	Yes	–	-	-	-	-	4,060	49	12.1	-	
**Knee extensor muscle strength**	Lowest	1,245	26	20.9	1.00 (reference)	<0.001	1,699	28	16.5	1.00 (reference)	<0.001
	Middle	1,243	12	9.7	0.46 (0.23–0.91)		1,700	14	8.2	0.50 (0.26–0.95)	
	Highest	1,243	7	5.6	0.27 (0.16–0.61)		1,699	7	4.1	0.25 (0.11–0.57)	
**Cardiorespiratory fitness**	Lowest	1,243	24	19.3	1.00 (reference)	0.045	1,699	21	12.3	1.00 (reference)	0.238
	Middle	1,245	14	11.2	0.58 (0.23–1.48)		1,698	17	10.0	0.81 (0.37–1.79)	
	Highest	1,243	7	5.6	0.33 (0.11–1.02)		1,701	11	6.5	0.53 (0.18–1.58)	

CI, confidence interval.
[a] Prevalence per 1,000 persons.

eTable 3. Associations of knee extensor muscle strength or cardiorespiratory fitness with low bone stiffness (YAM70%)

	Knee extensor muscle strength			*P* for trend
	Lowest	Middle	Highest	
**Men**				
Age adjusted	1.00 (reference)	0.50 (0.25–1.00)	0.32 (0.14–0.74)	0.004
Multivariable adjusted[a]	1.00 (reference)	0.47 (0.22–0.98)	0.22 (0.14–0.35)	<0.001
Mutual adjusted[b]	1.00 (reference)	0.47 (0.23–0.97)	0.23 (0.01–0.55)	<0.001
**Women**				
Age adjusted	1.00 (reference)	0.83 (0.43–1.61)	0.72 (0.30–1.72)	0.416
Multivariable adjusted[a]	1.00 (reference)	0.61 (0.31–1.20)	0.41 (0.18–0.95)	0.035
Mutual adjusted[b]	1.00 (reference)	0.61 (0.31–1.20)	0.42 (0.17–1.02)	0.037
**Post-menopausal**				
Age adjusted	1.00 (reference)	0.83 (0.59–1.18)	0.72 (0.30–1.72)	0.420
Multivariable adjusted[a]	1.00 (reference)	0.61 (0.32–1.19)	0.41 (0.18–0.97)	0.035
Mutual adjusted[b]	1.00 (reference)	0.61 (0.31–1.20)	0.42 (0.17–1.02)	0.037
	**Cardiorespiratory fitness**			***P* for trend**
	**Lowest**	**Middle**	**Highest**	
**Men**				
Age adjusted	1.00 (reference)	0.62 (0.24–1.60)	0.38 (0.12–1.20)	0.085
Multivariable adjusted[a]	1.00 (reference)	0.54 (0.33–0.89)	0.24 (0.13–0.44)	0.016
Mutual adjusted[b]	1.00 (reference)	0.56 (0.34–0.91)	0.26 (0.14–0.48)	0.022
**Women**				
Age adjusted	1.00 (reference)	1.40 (0.60–3.27)	1.40 (0.43–4.60)	0.504
Multivariable adjusted[a]	1.00 (reference)	1.23 (0.51–2.96)	1.05 (0.30–3.69)	0.876
Mutual adjusted[b]	1.00 (reference)	1.27 (0.53–3.04)	1.07 (0.31–3.63)	0.849
**Post-menopausal**				
Age adjusted	1.00 (reference)	1.37 (0.59–3.20)	1.40 (0.43–4.55)	0.516
Multivariable adjusted[a]	1.00 (reference)	1.23 (0.51–2.96)	1.05 (0.30–3.69)	0.874
Mutual adjusted[b]	1.00 (reference)	1.27 (0.53–3.04)	1.07 (0.31–3.63)	0.849

Values were expressed as odds ratio (95% confidence interval)

[a] additionally adjusted smoking status, drinking habits, BMI, systolic blood pressure, and menopause (Women only).

[b] mutually adjusted cardiorespiratory fitness or knee extensor muscle strength plus the variables above.

eTable 4. Interacting association of knee extensor muscle strength and cardiorespiratory fitness with low bone stiffness (YAM70%)

	Participant	Frequency	Prevalence[a]	Odds ratio (95% CI)[b]	Combined Odds ratio (95% CI)[c]	*P* for interaction
**Men**						0.985
**Lowest** cardiorespiratory fitness						
**Lowest** knee extensor muscle strength	719	16	22.3	1.00 (reference)	1.00 (reference)	
**Middle** knee extensor muscle strength	688	8	11.6	0.55 (0.45–0.66)	0.55 (0.21–1.42)	
**Highest** knee extensor muscle strength	484	3	6.2	0.22 (0.16–0.30)	0.21 (0.05–0.91)	
**Highest** cardiorespiratory fitness						
**Lowest** knee extensor muscle strength	551	10	18.1	1.00 (reference)	0.63 (0.20–2.00)	
**Middle** knee extensor muscle strength	566	4	7.1	0.37 (0.26–0.44)	0.22 (0.06–0.82)	
**Highest** knee extensor muscle strength	766	4	5.2	0.25 (0.20–0.33)	0.16 (0.05–0.54)	
**Women**						0.521
**Lowest** cardiorespiratory fitness						
**Lowest** knee extensor muscle strength	1,037	20	19.3	1.00 (reference)	1.00 (reference)	
**Middle** knee extensor muscle strength	902	8	8.9	0.47 (0.39–0.57)	0.46 (0.18–1.17)	
**Highest** knee extensor muscle strength	612	2	3.3	0.37 (0.28–0.49)	0.35 (0.08–1.48)	
**Highest** cardiorespiratory fitness						
**Lowest** knee extensor muscle strength	663	9	13.6	1.00 (reference)	0.85 (0.25–2.94)	
**Middle** knee extensor muscle strength	798	6	7.5	0.95 (0.75–1.21)	0.81 (0.27–2.45)	
**Highest** knee extensor muscle strength	1,088	4	3.7	0.48 (0.36–0.63)	0.42 (0.11–1.63)	
**Post-menopausal**						0.521

**Lowest** cardiorespiratory fitness					
**Lowest** knee extensor muscle strength	958	11	11.5	1.00 (reference)	1.00 (reference)
**Middle** knee extensor muscle strength	765	6	7.8	0.48 (0.19–1.22)	0.47 (0.18–1.18)
**Highest** knee extensor muscle strength	441	2	4.5	0.36 (0.08–1.57)	0.34 (0.08–1.47)
**Highest** cardiorespiratory fitness					
**Lowest** knee extensor muscle strength	592	2	3.4	1.00 (reference)	0.81 (0.23–2.78)
**Middle** knee extensor muscle strength	603	5	8.3	1.01 (0.27–3.78)	0.82 (0.27–2.47)
**Highest** knee extensor muscle strength	701	3	4.3	0.49 (0.12–2.09)	0.42 (0.11–1.64)

CI, confidence interval.

[a] Prevalence per 1,000 persons.

[b] Adjusted for age, smoking status, drinking habits, body mass index, and menopause (Women only).

[c] Using lowest cardiorespiratory fitness and lowest knee extensor muscle strength as reference.

eTable 5. Univariate associations of each covariate and two main exposure variables with low bone stiffness (complete case)

		Men					Women				
**Variables**	**Units**	**Participant**	**Frequency**	**Prevalence[a]**	**Odds ratio (95% CI)**	***P* value**	**Participant**	**Frequency**	**Prevalence[a]**	**Odds ratio (95% CI)**	***P* value**
**Age**	Years	2,235	241	107.8	1.01 (0.99–1.03)	0.070	2,890	499	172.7	1.10 (1.08–1.11)	<0.001
**Smoking status**	No	1,987	197	99.1	1.00 (reference)	<0.001	2,787	480	172.2	1.00 (reference)	0.747
	Yes	248	44	177.4	1.96 (1.37–2.80)		103	19	184.5	1.09 (0.65–1.81)	
**Drinking habits**	No	725	94	129.7	1.00 (reference)	0.022	1,998	382	191.2	1.00 (reference)	<0.001
	Yes	1,510	147	97.4	0.72 (0.55– 0.95)		892	117	131.2	0.64 (0.51–0.80)	
**Body mass index**	<18.5	74	11	148.6	1.43 (0.74– 2.76)	0.566	294	81	275.5	1.77 (1.34– 2.34)	0.004
	18.5–25.0	1,641	179	109.1	1.00 (reference)		2,000	354	177.0	1.00 (reference)	
	≥25.0	520	51	98.1	0.88 (0.64– 1.23)		596	64	107.4	0.56 (0.42–0.74)	
**Menopause**	No	-	-	-	-	-	498	10	20.1	1.00 (reference)	<0.001
	Yes	-	-	-	-	-	2,382	489	205.3	12.86 (6.83–24.25)	
**Knee extensor muscle strength**	Lowest	743	113	152.1	1.00 (reference)	<0.001	958	210	219.2	1.00 (reference)	<0.001
	Middle	748	72	96.3	0.59 (0.43–0.81)		973	174	178.8	0.78 (0.62–0.97)	
	Highest	744	56	75.3	0.45 (0.32–0.64)		959	115	119.9	0.49 (0.38–0.62)	
**Cardiorespiratory fitness**	Lowest	755	102	135.1	1.00 (reference)	<0.001	981	207	211.0	1.00 (reference)	0.231
	Middle	740	86	116.2	0.84 (0.62–1.14)		972	173	178.0	0.81 (0.65–1.01)	
	Highest	740	53	71.6	0.49 (0.35–0.70)		937	119	127.0	0.54 (0.43 –0.70)	

CI, confidence interval.
[a] Prevalence per 1,000 persons.

eTable 6. Associations of knee extensor muscle strength or cardiorespiratory fitness with low bone stiffness (complete case)

	Knee extensor muscle strength			*P* for trend
	Lowest	Middle	Highest	
**Men**				
Age adjusted	1.00 (reference)	0.58 (0.41–0.80)	0.43 (0.29–0.63)	<0.001
Multivariable adjusted†	1.00 (reference)	0.57 (0.40–0.80)	0.39 (0.26–0.59)	<0.001
Mutual adjusted§	1.00 (reference)	0.59 (0.42–0.83)	0.41 (0.27–0.62)	<0.001
**Women**				
Age adjusted	1.00 (reference)	1.09 (0.86–1.38)	1.00 (0.76–1.32)	0.911
Multivariable adjusted†	1.00 (reference)	0.89 (0.69–1.13)	0.73 (0.55–0.97)	0.034
Mutual adjusted§	1.00 (reference)	0.99 (0.69–1.13)	0.74 (0.55–0.99)	0.046
**Pre-menopausal**				
Age adjusted	1.00 (reference)	1.78 (0.08–39.66)	4.26 (0.22–81.26)	0.199
Multivariable adjusted†	1.00 (reference)	0.86 (0.04–20.80)	0.92 (0.04–20.05)	0.998
Mutual adjusted§	1.00 (reference)	0.86 (0.04–19.94)	0.81 (0.04–19.74)	0.981
**Post-menopausal**				
Age adjusted	1.00 (reference)	1.08 (0.85–1.37)	0.99 (0.75–1.30)	0.984
Multivariable adjusted†	1.00 (reference)	0.89 (0.70–1.14)	0.71 (0.53–0.96)	0.027
Mutual adjusted§	1.00 (reference)	0.89 (0.70–1.14)	0.72 (0.54–0.97)	0.034

	Cardiorespiratory fitness			*P* for trend
	Lowest	Middle	Highest	
**Men**				
Age adjusted	1.00 (reference)	0.84 (0.61–1.16)	0.49 (0.33–0.73)	<0.001
Multivariable adjusted[a]	1.00 (reference)	0.75 (0.54–1.05)	0.40 (0.26–0.60)	<0.001
Mutual adjusted[b]	1.00 (reference)	0.78 (0.56–1.08)	0.42 (0.27–0.63)	<0.001
**Women**				
Age adjusted	1.00 (reference)	1.37 (1.07–1.75)	1.33 (1.00–1.77)	0.032
Multivariable adjusted[a]	1.00 (reference)	1.13 (0.88–1.46)	0.93 (0.69–1.26)	0.974
Mutual adjusted[b]	1.00 (reference)	1.14 (0.89–1.47)	0.96 (0.71–1.31)	0.911
**Pre-menopausal**				
Age adjusted	1.00 (reference)	3.10 (0.41–23.31)	1.13 (0.13–10.02)	0.791
Multivariable adjusted[a]	1.00 (reference)	2.48 (0.33–18.50)	0.45 (0.06–3.72)	0.218
Mutual adjusted[b]	1.00 (reference)	2.52 (0.33–19.25)	0.47 (0.06–3.96)	0.232
**Post-menopausal**				
Age adjusted	1.00 (reference)	1.27 (0.99–1.62)	1.31 (0.98–1.74)	0.051
Multivariable adjusted[a]	1.00 (reference)	1.12 (0.87–1.45)	0.96 (0.71–1.31)	0.906
Mutual adjusted[b]	1.00 (reference)	1.13 (0.87–1.46)	1.00 (0.74–1.36)	0.911

Values were expressed as odds ratio (95% confidence interval)
[a] additionally adjusted smoking status, drinking habits, BMI, and menopause (Women only).
[b] mutually adjusted cardiorespiratory fitness or knee extensor muscle strength plus the variables listed above.

eTable 7. Interacting association of knee extensor muscle strength and cardiorespiratory fitness with low bone stiffness (complete case)

	Participant	Frequency	Prevalence[a]	Odds ratio (95% CI)[b]	Combined Odds ratio (95% CI)[c]	*P* for interaction
**Men**						0.489
**Lowest** cardiorespiratory fitness						
**Lowest** knee extensor muscle strength	544	91	167.3	1.00 (reference)	1.00 (reference)	
**Middle** knee extensor muscle strength	377	41	108.8	0.46 (0.29–0.71)	0.57 (0.37–0.87)	
**Highest** knee extensor muscle strength	208	20	96.2	0.32 (0.18–0.58)	0.39 (0.22–0.68)	
**Highest** cardiorespiratory fitness						
**Lowest** knee extensor muscle strength	199	22	110.6	1.00 (reference)	0.48 (0.28–0.81)	
**Middle** knee extensor muscle strength	371	31	83.6	0.65 (0.35–1.19)	0.31 (0.19–0.52)	
**Highest** knee extensor muscle strength	536	36	67.2	0.60 (0.31–1.13)	0.23 (0.13–0.39)	
**Women**						0.398
**Lowest** cardiorespiratory fitness						
**Lowest** knee extensor muscle strength	656	147	224.1	1.00 (reference)	1.00 (reference)	
**Middle** knee extensor muscle strength	529	107	202.3	0.81 (0.59–1.10)	0.88 (0.65–1.20)	
**Highest** knee extensor muscle strength	287	41	142.9	0.70 (0.46–1.06)	0.78 (0.51–1.18)	
**Highest** cardiorespiratory fitness						
**Lowest** knee extensor muscle strength	302	63	208.6	1.00 (reference)	1.05 (0.73–1.51)	
**Middle** knee extensor muscle strength	404	67	165.8	0.96 (0.63–1.44)	0.93 (0.64–1.34)	
**Highest** knee extensor muscle strength	672	74	110.1	0.83 (0.54–1.26)	0.72 (0.50–1.06)	
**Post-menopausal**						0.563
**Lowest** cardiorespiratory fitness						
**Lowest** knee extensor muscle strength	618	146	236.2	1.00 (reference)	1.00 (reference)	
**Middle** knee extensor muscle strength	471	106	225.1	0.81 (0.59–1.11)	0.89 (0.66–1.21)	

**Highest** knee extensor muscle strength	223	40	179.4	0.70 (0.46–1.08)	0.77 (0.51–1.18)
**Highest** cardiorespiratory fitness					
**Lowest** knee extensor muscle strength	279	63	225.8	1.00 (reference)	1.06 (0.74–1.53)
**Middle** knee extensor muscle strength	347	66	190.2	0.95 (0.63–1.44)	0.94 (0.65–1.37)
**Highest** knee extensor muscle strength	444	68	153.2	0.80 (0.52–1.23)	0.71 (0.48–1.04)

CI, confidence interval.

[a] Prevalence per 1,000 persons.

[b] Adjusted for age, smoking status, drinking habits, body mass index, and menopause (Women only).

[c] Using lowest cardiorespiratory fitness and lowest knee extensor muscle strength as reference.
